# Temperature Effects on Electromechanical Response of Deposited Piezoelectric Sensors Used in Structural Health Monitoring of Aerospace Structures

**DOI:** 10.3390/s19122805

**Published:** 2019-06-22

**Authors:** Hamidreza Hoshyarmanesh, Mojtaba Ghodsi, Minjae Kim, Hyung Hee Cho, Hyung-Ho Park

**Affiliations:** 1 Project neuroArm, Health Research Innovation Center, University of Calgary, Calgary, AB T2N 4Z6, Canada; hamidreza.hoshyarman@ucalgary.ca; 2 Department of Mechanical and Industrial Engineering, Sultan Qaboos University, Muscat 123, Sultanate of Oman; ghodsi@squ.edu.om; 3 Department of Materials Science and Engineering, Yonsei University, Seoul 03722, Korea; minjae_kim@yonsei.ac.kr; 4 School of Mechanical Engineering, Yonsei University, Seoul 03722, Korea; hhcho@yonsei.ac.kr

**Keywords:** piezoelectric sensor, lead zirconate titanate/lead zirconate titanate (PZT/PZT), lead zirconate titanate/bismuth titanate (PZT/BiT), temperature effect, electromechanical impedance, aerospace structure, superalloy blade, structural health monitoring, damage detection

## Abstract

Turbomachine components used in aerospace and power plant applications preferably require continuous structural health monitoring at various temperatures. The structural health of pristine and damaged superalloy compressor blades of a gas turbine engine was monitored using real electro-mechanical impedance of deposited thick film piezoelectric transducers at 20 and 200 °C. IVIUM impedance analyzer was implemented in laboratory conditions for damage detection in superalloy blades, while a custom-architected frequency-domain transceiver circuit was used for semi-field circumstances. Recorded electromechanical impedance signals at 20 and 200 °C acquired from two piezoelectric wafer active sensors bonded to an aluminum plate, near and far from the damage, were initially utilized for accuracy and reliability verification of the transceiver at temperatures >20 °C. Damage formation in both the aluminum plate and blades showed a peak shift in the swept frequency along with an increase in the amplitude and number of impedance peaks. The thermal energy at 200 °C, on the other hand, enforces a further subsequent peak shift in the impedance signal to pristine and damaged parts such that the anti-resonance frequency keeps reducing as the temperature increases. The results obtained from the impedance signals of both piezoelectric wafers and piezo-films, revealed that increasing the temperature somewhat decreased the real impedance amplitude and the number of anti-resonance peaks, which is due to an increase in permittivity and capacitance of piezo-sensors. A trend is also presented for artificial intelligence training purposes to distinguish the effect of the temperature versus damage formation in sample turbine compressor blades. Implementation of such a monitoring system provides a distinct advantage to enhance the safety and functionality of critical aerospace components working at high temperatures subjected to crack, wear, hot-corrosion and erosion.

## 1. Introduction

Temperature effects have always been a critical problem for piezoelectric-based monitoring methods such as pitch-catch [1], pulse-echo [1,2] and electromechanical impedance spectroscopy (EMIS) [3,4,5,6,7,8], especially in detecting incipient damages. It has directed several research works towards the development or implementation of high-temperature piezoelectric transducers for structural health monitoring (SHM) of mechanical components, which work in harsh environments, and are subjected to wear, corrosion, erosion or surface cracking [9,10,11]. Li et al. [8] recently proposed the concept of a smart corrosion coupon for corrosion monitoring and studied the effect of temperature on the electromechanical impedance (EMI) signatures of PZT (lead zirconate titanate) patches at 10 to 40 °C. 

Permanently installed high-temperature piezoelectric wafer active sensors (HT-PWAS) have been one of the extensively employed sensor technologies for high-temperature SHM [12,13]. McNab et al. [14] used lithium niobate (LN) in 1998 as a high-temperature piezo-material with the Curie temperature of 1150 °C that could be used up to 600 °C. A report published by NASA [15] showed the temperature dependence of piezoelectric charge coefficients, effective electromechanical (E/M) coupling coefficient, thermal expansion and elastic compliance of three different piezoelectric materials. Park et al. [16] successfully used LN in an impedance-based monitoring system for damage detection of a bolted joint structure in the temperature range of 482–593 °C. Konstantinidis et al. [17] investigated the long-term temperature stability of a guided wave SHM system in a plate using permanently attached sensors and the optimal baseline subtraction (OBS) method that uses several baselines captured over a varying rate of temperature changes. They assessed the suitability and robustness of OBS and its use in damage localization using the pitch-catch technique. 

Kažys et al. [18], Akiyama et al. [19] and Krsmanovic [20] have compared piezoelectric properties and maximum working temperature of several piezoelectric materials. Giurgiutiu et al. [11] utilized gallium orthophosphate as a HT-PWAS at 260–705 °C, whereas, the piezoelectric properties of conventional PZT (lead zirconate titanate) declined at 260 °C and a paraelectric phase was observed at 370 °C (no impedance peaks). Bao [9] used langasite (LGS) piezoelectric material, glue-bonded to a Ti plate in a high-temperature environment from 20 to 700 °C, and E/M impedance spectrum to identify the possibility of developing transducers for crack detection at high-temperatures. LGS maintains its piezoelectric properties up to 700 °C, much higher than the maximum working temperature of conventional PZT (260 °C). Baptista et al. [21] investigated the effect of temperature on the E/M impedance signatures of a conventional 5H-PZT sensor used in SHM. The variations in both the amplitude and the frequency were analyzed experimentally by using an aluminum specimen and obtaining impedance signatures at temperatures ranging from 25 to 102 °C over a frequency range of 2–550 kHz. As piezoelectric sensors are predominantly capacitive devices, the variation of the capacitance with temperature was also analyzed at 26–67 °C. Koo et al. [22], Yun et al. [23] and Baptista et al. [21] used an effective frequency shift (EFS) based on shifting the updated impedance signature relative to the baseline signature to maximize the correlation coefficient and compensate for temperature effects. A test was also carried out by Balmès et al. [24] to analyze the correlation of temperature effects in piezoelectric materials and glue used in SHM by prediction of dynamic capacity and its evolution with temperature in both isolated and glued configurations. 

Li et al. [25] developed a high-temperature screen-printed BiScO_3_–xPbTiO_3_ (BS-PT) based piezoelectric transducer integrated with a thin polyimide (high-temperature polymer) substrate. Kamas [12] assessed the temperature effects on PWAS by utilizing the EMIS method in an increasing temperature environment up to 230 °C. The piezoelectric material degradation with temperature was investigated, and trends of variation with temperature were deduced from experimental measurements. Lissenden and Tittmann [13] sprayed sol-gel derived PZT/BT (lead zirconate titanate/bismuth titanate) and BT/LN (bismuth titanate/lithium niobate) piezoelectric transducers on a stainless steel pipe and used ultrasonic-guided waves to achieve high reliability for SHM service temperatures up to ~400 °C in nuclear power plants. Spray-on PZT/BT transducers were also fabricated and implemented by Malarich [26] in health monitoring of high-temperature structures. Eason et al. [27] also fabricated thin-film ceramic sensors using sol-gel technique for monitoring of steel pipes in oil industries subjected to high-temperature corrosion using pulse-echo and pitch-catch methods as well as time-of-flight approach and statistical analysis. Zhang et al. [28] studied a piezoelectric-based high-temperature crack detection system combined with a laser vibrometer mechanism (as a sensor for remote detection) for I-beams up to 150 °C. 

While several piezoelectric materials could be applicable for high-temperature applications [19], bismuth titanate (BiT) was selected by Prowant [29], given its relatively high E/M coupling coefficients and less degradation in sensitivity as the temperature is increased past ~400 °C. A major drawback with the prior studies is the lack of employment of such high-temperature transducers in field SHM of moving parts (e.g., aerospace structures or nuclear/steam/gas power plants at high speeds). Stacked PWAS transducers are regularly glue-bonded to the devices under test (DUT). The adhesive properties and its thickness could affect the sensor functionality and experiment data [9]. 

These challenges together with the shear lag, which occurs due to the adhesive layer, and detachment uncertainties of stacked sensors at high temperatures/high speeds, led us to replace PWAS by deposition of piezo-ceramic thick films on curved metallic substrates using sol-gel photochemical metal-organic method. For the past decade, the authors have been focused on the development of different integrated thin and composite thick piezoelectric films and characterizations [30,31,32,33,34,35], polarization process [36], and application of such films in the SHM of aerospace structures at ambient temperature and different rotary speeds [37]. This study, on the other hand, represents the effects of temperature variations on E/M impedance signatures of sol-gel derived deposited piezoelectric thick films deployed in SHM of aerospace structures. Investigation of and compensation for thermal effects are important in monitoring of such turbomachine structures that work at elevated temperatures. 

The paper has been organized as follows. A brief explanation of materials and methods is presented in Section 2. The experimental setup is explained in Section 3. Section 4 presents results and discussions. Section 5 is dedicated to the conclusions and future works.

## 2. Materials and Methods

Turbine engines are to be accounted as one of the crucial rotary structures in aerospace industries, for which the lack of appropriate continuous monitoring could likely cause catastrophic failures. Out of all components in a turbine engine, superalloy blades consist of as much as ~42% of the whole failures [38]. According to a statistical damage cost estimation for total aero-derivative turbines provided in [39], 24% of damage costs is assigned to the compressor surge and 7.5% for high cyclic fatigue (HCF) of compressor blades. In another study, the reason behind 50% of blade failures is associated with the stress corrosion cracking and corrosion fatigue due to excessive blade vibration [40]. Blades were recognized by Witos and Wachlaczenko [41] as critical components of a turbine engine, very prone to mechanical damages with a 55% probability of fatigue crack formation on the leading edge and 45% on the back surface. Apart from HCF, low cycle fatigue (LCF) and very high cycle fatigue (VHCF) of compressor and turbine blades were also introduced as serious challenges in SHM. Microcracks initiated at LCF and VHCF conditions inside the material at a normal level of the mechanical stress are difficult to detect by conventional non-destructive methods [41]. 

Logan [42] classified the compressor of a gas turbine engine as a turbomachine, subjected to LCF, HCF, VHCF, pitting and erosion wear [43]. Aligned with this classification, we have previously studied the SHM of compressor nickel-based superalloy blades, subjected to damage formation and loss in edge thickness as a result of stress conditions and erosion, caused by ingested large particles [37]. The working temperature and speed range of the compressor were 20–25 °C and 0–2000 rpm, respectively. However, the effect of high temperature was not considered in previous studies, as it only covered a semi-field working condition. This research significantly concentrates on the monitoring process of the compressor blades when operating at high speeds and temperatures within the standard ranges provided by Rolls Royce plc [44]. 

## 3. Experimental Setup

The hardware deployed for the present research is the same as the turbomachine prototype, previously designed and developed by the authors [37] with the capacity of working at 20–300 °C and 0–3000 r/min, consisting of six superalloy IN718 blades (engine JT8D; Pratt & Whitney, Longueuil, QC, Canada), named as L_1_–L_6_, mounted on an aluminum disk. (L_1_, L_4_), (L_2_, L_3_) and (L­_5_, L_6_) are the pairs of blades with 10 layers of PZT/BiT and PZT/PZT, and 5 layers of PZT/BiT thick films, respectively. The disk is driven via a stainless steel shaft and an electric motor. The blades have undergone ~5000 h of field operation in a gas turbine engine; however, they are still in good condition but not similar in terms of wear, homogeneity, thickness and tribology. The same portable transmitter-receiver as introduced in [33] was utilized to provide a frequency sweep range of 1 kHz–1 MHz for SHM in a moving condition and to record the EMI responses of the transducers. The transceiver is composed of an oscillator (1–1000 kHz), current and voltage amplifiers, a multiplexer, a current to voltage converter, a voltage to current converter, a phase detector, a peak detector, sensor connectors, a multimedia card (MMC), a liquid crystal display (LCD) screen, four Li-ion batteries, several voltage regulators, and a microcontroller. To stimulate the active sensors, a controllable signal generator (MAX038) with variable sweeping output frequency was also employed. The range and precision of the output frequency were adjusted using a variable capacitance and the V/A converter ratio. By measuring the frequency domain excitation signal *V_piezo_*(*f*), and response signal *I(f)* for all piezo-films, the impedance spectrum of the sensors was acquired, periodically, at intervals of every 12–15 min. This periodically activated approach was time efficient since there was no need to monitor the device under test continuously for a long time, which greatly reduced the lead time and power consumption. Rechargeable batteries were installed in a rotary housing block, specifically designed for structural health monitoring of moving/rotary critical industrial equipment working at up to 3000 rpm and 200 °C. 

The procedure of piezo-film deposition on the curved surface of the blades was similar to what was described in [31,34]. The samples went through gold bottom electrode deposition (600 nm per layer), prior to fabrication of thick film piezo-sensors. PZT precursor solution was prepared using lead 2-ethylhexanoate, zirconium 2-ethylhexanoate and Ti-isopropoxide in molar ratio Pb:Zr:Ti = 1.1:0.52:0.48 and hexane was used as a solvent. To make the BiT precursor solution Bi:Ti = 4.4:3, bismuth 2-ethylhexanoate was dissolved in hexane as the solvent. Then titanium isopropoxide was added and the mixture was stirred on the vibrator for 4 h. PZT/PZT and PZT/BiT composites were prepared with the introduction of a certain mass percentage of PZT-5A powder, with 2–5 µm particle size, in PZT and BiT precursor solutions, respectively. The resultant composite piezoelectric thick films were deposited on the curved surface of the blades up to 100 mm of thickness using sol–gel technique and were polarized as explained in [36]. All the samples were dried at 200 °C for 5 min, pyrolyzed at 400 °C for 5 min, UV irradiated and annealed at 600–700 °C in a tube furnace under atmosphere pressure for 30 or 60 min with a heating rate of 5 °C/min [31,34].

In this composition, the PZT element guarantees the high piezoelectric properties, while the BiT (*T_c_* = 550 °C) ensures high temperature performance of the sensors. Preliminary characterization of deposited piezo-films was also performed as reported in [31,32,34] to ensure the accurate structural composition and surface morphology, as well as mechanical, dielectric, ferroelectric and piezoelectric properties of the piezo-sensors. 

The entire hardware was accommodated within the interior space of a parabolic-shape nacelle, equipped with a tachometer, temperature sensor and a simple user-interface panel. The experiments were carried out at temperatures of 20, 100 and 200 °C and rotational speeds of 0 (static mode), 1000 and 2000 rpm. The blades were tested in pristine and damaged states. Upon completion of studying the pristine blades and recording the baseline signatures of the E/M impedance, a longitudinal rectangular-shaped notch of the size 15 × 0.7 mm was formed on the leading edge of each blade, as described in [33,37]. The notch was machined to a depth of 2 mm, where it met the central cooling conduit of the blade. Comparing to a baseline at each condition helps lift the adverse impacts of uncertainties. High temperatures usually enhance and speed up damage formation. Nevertheless, damage due to fatigue, corrosion and erosion occurs also at 20–200 °C in many stationary and moving structures. Hence, the temperature was limited to 200 °C in the present study, considering the safety issues of laboratory experiments. To ensure the uniformity of the temperature on the top surface of the DUTs, in the neighborhood zone of the active measuring sensors, we used an infrared thermometer with laser sighting, model HN235. Using a continuous temperature feedback in the measurement system and considering the heat dissipation rate in laboratory conditions, the hotplate temperature was adjusted, appropriately.

Measurement of the E/M impedance of the sensors was performed in stationary mode. An IVIUM impedance analyzer, a developed portable transceiver, a hot plate, laptop and Matlab^®^ (software) were utilized. IVIUM analyzer was deployed as a standard reference device to calibrate the custom-built transceiver. All the experiments were repeated at least three times and the average quantities were reported. 

**Figure 1:**
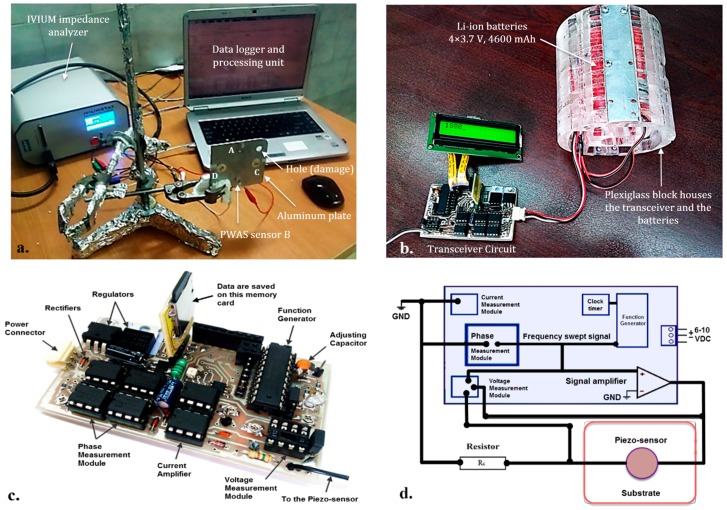
Hardware setup for the electromechanical impedance (EMI) frequency response measurement of bonded piezoelectric wafer active sensors (PWAS) sensors to an aluminum plate (100 × 100 mm), using (**a**) IVIUM analyzer and (**b**) transceiver circuit. (**c**) Portable EMI-based transceiver circuit and (**d**) architecture of transceiver components.

A series of experiments were initially conducted on an aluminum plate with two sets of sensors {A,B} and {C,D}. Two PWASs pairs {A,B}, with diameter = 10 mm, thickness = 1 mm, and {C,D} with diameter = 17 mm and thickness = 0.2 mm, were deployed across the plate. A circular hole of 10 mm in diameter, drilled at the top right corner of the plate, played the role of the damage. Sensors {A,B} were located vertically closer and farther to the damage as described in [33]. Similar layout was applied for sensors {C,D}. Both the IVIUM and the portable transceiver, shown in Figure 1, were used to investigate the EMI signatures of four PWASs bonded to the surface of the aluminum plate at room temperature (20 °C) and a higher temperature (200 °C). The frequency (*f*) was swept in logarithmic scale for the IVIUM analyzer, and no peaks were observed at *f* > 500 kHz. The frequency swept by the transceiver circuit was linearly spanned from 1 kHz to 1.7 MHz.

**Figure 2:**
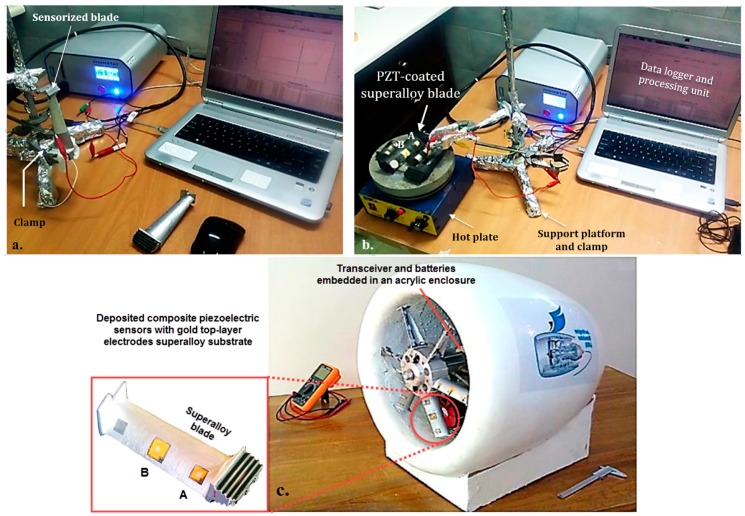
Hardware setup for the EMI frequency response measurement of deposited piezo-films on superalloy blades at (**a**) 20 °C and (**b**) 200 °C. (**c**) The turbomachine developed for semi-field tests.

The experiments were repeated, similarly, for six superalloy blades to measure the frequency response of deposited piezo-films using IVIUM impedance analyzer at 20 and 200 °C (Figure 2). The EMIS of the deposited piezo-films was primarily recorded in the pristine state. Consequently, the EMIS was also recorded for the same blades in a damaged state. A defect of 50 × 1 mm slot on the leading edge formed a sort of conventional damage, which generally occurs in the compressor blades. At 20 °C, the roots and shrouds of the blades were clamped to a stand and the sensors were hardwired to the measuring system. At higher temperatures, the blades were laid on the hot plate. The high frequency output signals obtained from piezo-sensors are not normally affected by supporting methods as the local nanoscale deflections due to piezoelectric stimulation are negligible compared to the dimensions, mass and stiffness of the blade. The used frequency bandwidth is also much higher than that affected by low frequency mechanical uncertainties. 

## 4. Results and Discussions

### 4.1. Health Monitoring of Aluminum Plate

**Figure 3:**
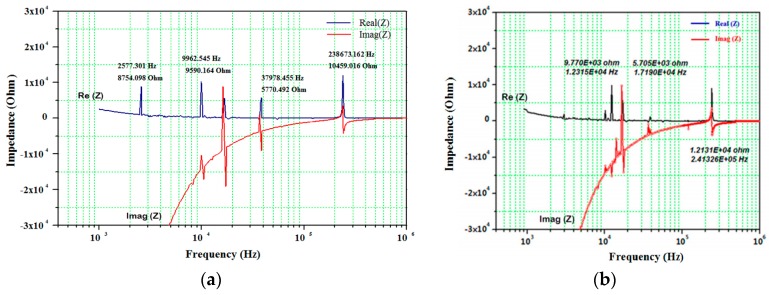
EMI frequency spectrum of sensor A bonded to the aluminum plate at (**a**) 200 °C, and (**b**) 20 °C using the transceiver (flat segments with no peaks have been neglected).

Real (*Z*_1_) and imaginary (*Z*_2_) parts of impedance frequency spectrum for the sensor A bonded to the aluminum plate, closer to the hole, was recorded using IVIUM analyzer and transceiver circuit at 20 °C [33,37] and 200 °C. Focusing on the ambient temperature and investigating the effect of the damage formation in the structure, as reported in [33,34], sensor A showed a peak frequency shift, higher anti-resonant peaks and an increased number of peaks over the entire measured frequency band compared to sensor B. The above differences in EMI spectrums were in good agreement between the results taken from IVIUM analyzer and transceiver circuit at ambient temperature for the stationary SHM of a metal plate specimen. By increasing the temperature, the EMI signature was measured and the average of three measurements was also recorded at 200 °C. As shown in Figure 3, the corresponding peak frequencies for sensor A at 200 °C vs. 20 °C using the transceiver are designated as 9.962 vs. 12.31 kHz, and 238.673 vs. 241.32 kHz. The EMI amplitude for these peaks were recorded as 9.59 vs. 9.77 kΩ, and 10.46 vs. 12.131 kΩ. A sensible reduction in EMI amplitude is detected at anti-resonance frequencies too. 

### 4.2. Health Monitoring of Superalloy Blades

**Figure 4:**
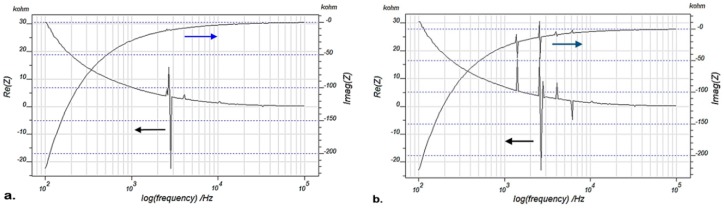
Real and imaginary EMI for the blade L_1_, frequency sweep at 100 Hz–100 kHz using IVIUM analyzer at 20 °C; (**a**) pristine blade and (**b**) damaged blade.

**Figure 5:**
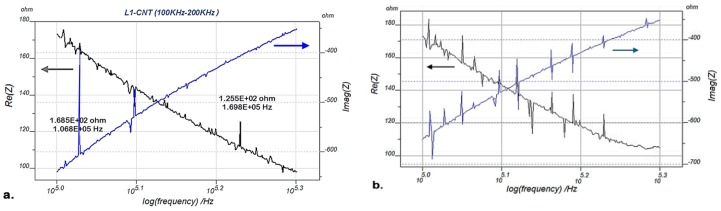
Real and imaginary EMI for the blade L_1_, frequency sweep at 100–200 kHz using IVIUM analyzer at 20 °C; (**a**) pristine blade and (**b**) damaged blade.

**Figure 6:**
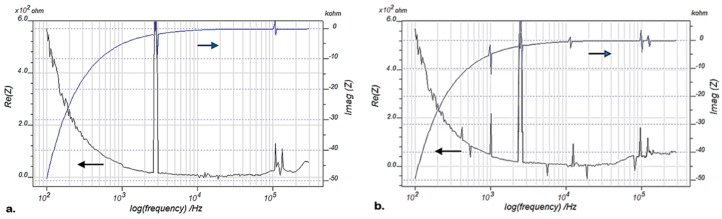
Real and imaginary EMI for the blade L_1_, frequency sweep at 100 Hz–300 kHz using IVIUM analyzer at 200 °C; (**a**) pristine blade and (**b**) damaged blade.

EMI signatures of the sample blade L_1_ (PZT/BiT) are presented in Figure 4, Figure 5 and Figure 6 at pristine and damaged states. Figure 4 shows the real and imaginary parts of EMI for the blade L_1_ with a frequency sweep from 100 Hz to 100 kHz, using IVIUM analyzer at 20 °C. Figure 5 illustrates the same parameters measured for the blade L_1_ at a wider bandwidth of 100–200 kHz at 20 °C. Figure 6 depicts the real and imaginary parts of EMI for the blade L_1_, swept at 100 Hz–300 kHz at 200 °C using IVIUM analyzer.

Table 1 summarizes the E/M frequency response of the sensors bonded to the pristine and damaged blades L_1_ and L_3_ (PZT/PZT) at 20 and 200 °C. An obvious difference is detected between the two blades in terms of EMI amplitude and frequency shift at anti-resonance conditions. Temperature variation further reduces the impedance and anti-resonance frequencies. 

**Figure 7:**
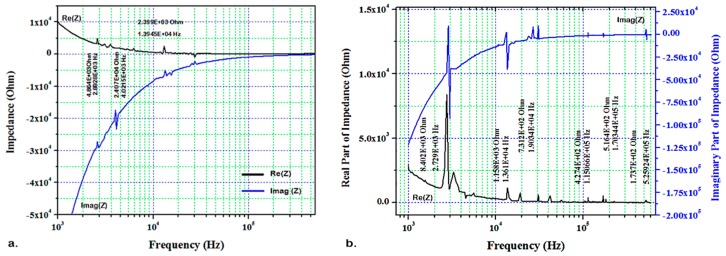
Real and imaginary EMI for the blade L_2_, frequency sweep at 1–600 kHz using transceiver board at 20 °C; (**a**) pristine blade and (**b**) damaged blade.

**Figure 8:**
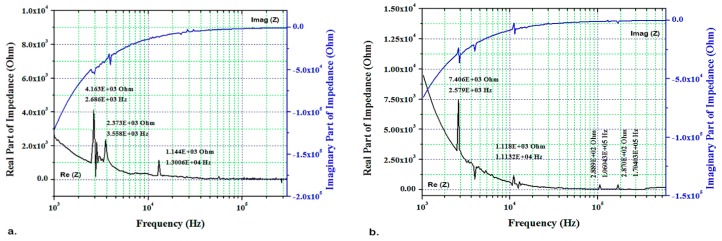
Real and imaginary EMI for the blade L_2_, frequency sweep at 1–600 kHz using the transceiver circuit at 200 °C; (**a**) pristine blade and (**b**) damaged blade.

The experiments were repeated using the transceiver circuit with the same conditions as measured by IVIUM. Thus, calibration coefficients were derived at 1–600 kHz. The real and imaginary parts of EMI for the pristine and damaged blade L_2_ (PZT/PZT) at ambient temperature are presented in Figure 7 at 1 to 600 kHz. Figure 8 shows the same parameters for the blade L_2_ using the transceiver circuit at 200 °C. The results for the peak values obtained from Figure 7 and Figure 8 are compared in Table 2. 

Table 2 shows the E/M frequency response of the sensors bonded to the pristine and damaged blades L_2_ (PZT/PZT) and L_4_ (PZT/BiT) at 20 and 200 °C, measured by the transceiver circuit. The results acquired by the transceiver also reveal (i) a left shift for the peak frequencies, (ii) an increase in the EMI amplitude and (iii) a higher number of peaks (local resonances) in the damaged blade (Figure 7b and Figure 8b) compared to the pristine state (Figure 7a and Figure 8a) that confirm the results obtained by IVIUM analyzer. 

The effect of temperature on the abovementioned parameters is evident by comparing Figure 7 and Figure 8, which turns to the fact that increasing the temperature will shift the peak frequencies of the damaged blades even further to the left. Therefore, the minimum peak frequency was recorded for the damaged blade at 200 °C, which undertakes the effects of both the mechanical dislocations and thermal energy. Another effect of the temperature increase is a sensible reduction in the EMI amplitude. Damage formation turns normally into an increase in the EMI amplitude, which makes the peak amplitude of a sample at a pristine state less than of that at the damaged state. 

Increasing the temperature stretched the difference between these two states even further such that the minimum peak amplitude was recorded for the pristine blade at 200 °C. The third effect of the temperature is disappearance of some peaks which is related to a change in the damping ratio and local resonance mode shapes [7]. The obtained results in this research are in good agreement with what Baptista [21] and Li [8] reported in 2014 and 2019, respectively. The frequency shifts of the E/M peaks increased over the entire frequency range. In addition, increasing the temperature from 20 to 200 °C produced a considerable frequency decrease as mentioned in Table 3. 

The results at ambient temperature confirm a left-shift (reduction) in the E/M anti-resonance frequencies ($↓\omega_{n}=\sqrt{k/m}$) of piezoelectric sensors in damaged structures due to damage formation. It is also evident that the amplitude of peaks in damaged samples is larger and their number increases compared to pristine ones. The real part of EMI in damaged blades shows larger peaks than that of pristine blades at the same temperature.

Increasing the temperature to 200 °C mitigates the anti-resonance EMI frequencies of deposited piezo-films in damaged substrates, as shown in Figure 6 and Table 1, which is in good agreement with [9,21]. This could be interpreted as a decrease in the dynamic stiffness of the sensor (${\bar{k}}_{s}$) due to an increase in the complex mechanical compliance (${\bar{s}}_{11}^{E}$) of the piezoelectric film as given by Equation (1) [45,46]. Increasing the temperature also affects the local stiffness of the substrate at the vicinity of the sensor ($↓k_{str}\propto ↓E_{str}$) [11,46].

(1) $$↓{\bar{k}}_{s}\propto 1/\left({\bar{s}}_{11}^{E}↑\right)$$

The second effect of the temperature is a reduction in the amplitude of the anti-resonant peaks. As reported in [7,45], the frequency-dependent EMI of a piezo-sensor constrained to a substrate could be modeled as Equation (2):

(2) $$\bar{Z}(\omega )=\frac{1}{i\omega \bar{C}}{\left[1-{\bar{K}}_{31}^{2}\frac{Z_{str}(\omega )}{Z_{str}(\omega )+Z_{A}(\omega )}\right]}^{-1}$$

Where, $\bar{C}$ is the lossy capacitance, ${\bar{K}}_{31}^{}$ is the lossy piezoelectric coupling factor, $Z_{str}(\omega )$ is the frequency-dependent local mechanical impedance of the structure, and $Z_{A}(\omega )$ stands for the frequency-dependent electrical impedance of the sensor. With respect to the fact that the modulus of elasticity of the piezoelectric sensors decreases by increasing the temperature [47] and assuming a relationship between the complex mechanical compliance of the sensor at high temperatures (e.g., 200 °C ($s_{11}^{HT}$)), and the mechanical compliance at ambient temperature ($s_{11}^{LT}$), it could be concluded that there is an increase in the thermal damping ratio of the sensor ($\eta_{T}↑$) as temperature increases. This is shown by Equation (3).

(3) $$s_{11}^{HT}=s_{11}^{LT}(1-i\eta_{T}),\;s_{11}^{HT}>s_{11}^{LT}$$

On the other hand, the IN718 substrates used in this research have been used for more than five years in a turbine engine. Hence, they were significantly strained both thermally and mechanically. For a strained sample of IN718, the internal friction or mechanical loss ($Q^{-1}$) increases slightly by increasing the temperature from 20 to 200 °C, as reported in [48]. Increasing the internal friction due to interaction between dislocations and defects induced by stress, means the damping ratio of the substrate also slightly increased. Equation (4) presents the variation of the substrate damping ratio with respect to the changes in structural and critical damping of the structure.

(4) $$↑\xi_{str}=\frac{↑c_{str}}{2m\omega_{n}↓}$$

**Figure 9:**
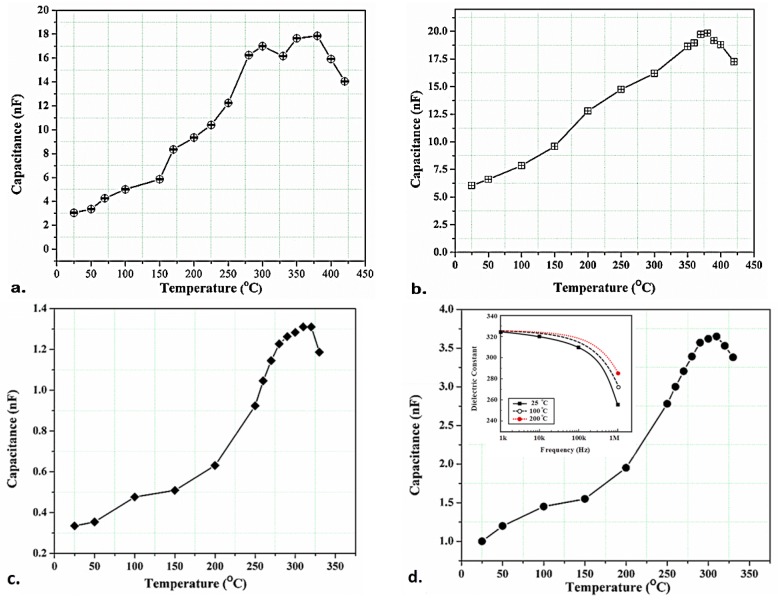
Capacitance variation for: (**a**) PZT/BiT on L_1_ (location A), (**b**) PZT/BiT on L_1_ (location B), (**c**) PZT/PZT on L_3_ (location A), and (**d**) PZT/PZT on L_3_ (location B).

The reduction in the amplitude as well as left frequency shift of the real part, the imaginary part and the magnitude of the impedance signatures, by increasing the temperature, are also potentially related to the temperature-dependence of the permittivity (dielectric constant) and, as a result, the capacitance of the piezoelectric sensor. Dielectric permittivity and capacitance of the sensors increase as the temperature increases to 200 °C, as shown in Figure 9. A slight increase in the complex electrical damping ratio ($\delta_{T}$) may cause such an increase in the permittivity and capacitance as presented by Equation (5).

(5) $$\varepsilon_{33}^{HT}=\varepsilon_{33}^{LT}(1-i\delta_{T}),\;\varepsilon_{33}^{HT}>\varepsilon_{33}^{LT}$$

Due to the increase in $\varepsilon_{33}^{E}$ and $s_{11}^{E}$, there would be a resultant reduction in the coupling factor, as yielded by Equation (6):

(6) $$↓{\bar{K}}_{31}^{2}=\frac{d_{31}^{2}}{{\bar{s}}_{11}^{E}↑\;{\bar{\varepsilon }}_{33}^{E}↑}$$

Similarly, there would be an increase in the sensor capacitance as given by $↑\bar{C}\propto {\bar{\varepsilon }}_{33}^{E}↑$. These all result in observing a reduction in the E/M impedance peaks at antiresonance frequencies at 200 °C. Figure 9a,b shows capacitance variation of the piezo-sensors PZT/BiT deposited on the locations A and B of the blade L_1_ (A and B are near the root and in the middle of the IN718 blade’s edge, respectively), with the temperature changing from 25 to 420 °C. Figure 9c,d, on the other hand, shows the same parameter change for the piezo-sensors PZT/PZT deposited at A and B of the blade L_3_, with the temperature changing from 25 to 330 °C. The diagrams confirm a continuous increase in the capacitance of the piezoelectric thick films as the temperature increases from 25 to 200 °C. This increase has a significant influence on the EMI amplitude as clearly stated in [21]. The inset of Figure 9d also illustrates the frequency dependency of the dielectric constant at different temperatures: 25, 100 and 200 °C. It indicates that dielectric constant decreases drastically at high frequencies (>100 kHz) and lower temperatures intensify the rate of dielectric change. 

The third effect of the temperature is the appearance of new peaks due to formation of new local vibration modes. The increase in temperature shows similar effects to damage formation (at low temperatures) on the variation of stiffness and anti-resonance frequencies. As reported in [7,45], variations in the stiffness of both the sensor and the substrate, as well as change in the damping ratio would cause the formation of new peaks. In the case of temperature increase, several new peaks are displayed in each graph at 200 °C, which we think may or may not be the result of a left shift to the anti-resonance peaks. These new peaks appear at frequencies lower than those ones that were expected to appear in low temperatures.

An effective frequency shift (EFS) algorithm together with machine learning technique is being used by the authors to compensate the temperature effects on the output signals. The data provided in Table 1, Table 2 and Table 3 have been implemented as a trend. To eliminate the effect of temperature, it is required to measure a baseline for the impedance frequency spectrum of the pristine blade at the same temperature. The signatures obtained at different temperatures and damage severity levels with the corresponding baselines at normal conditions (i.e., ambient temperature and pristine-state, respectively), would help distinguish the effect of the temperature versus damage formation in turbine compressor blades. 

## 5. Conclusions

Superalloy blades of a gas turbine compressor were structurally monitored in pristine and damaged states by investigation of the real EMI of deposited thick film piezoelectric sensors at 20 and 200 °C. Experiments were conducted by implementation of an aluminum plate and several superalloy blades, consecutively. Calibration of a custom-built transceiver board was performed at this stage using a reference IVIUM analyzer. The real part of the EMI signatures was recorded and thermally classified as pristine state versus damaged state and Re(*Z*) at 20 °C versus Re(*Z*) at 200 °C. The overall results showed that when damage occurs, peak frequencies shift towards less values, whereas there is a jump in the EMI amplitude and number of peaks (local resonances). Meanwhile, as the temperature increases, a further left shift is obvious in Re(*Z*) along with a sensible reduction in the EMI amplitude and number of peaks, which are related to change in the permittivity and capacitance of piezo-sensors. Recording a baseline graph for each part at ambient temperature and pristine state would result in a successful and compensable continuous SHM of aerospace structures, which are exposed to crack, wear, corrosion or erosion at high temperatures.

## Figures and Tables

**Table 1:** Anti-resonance frequency of the blades L_1_ and L_3_ at 20 and 200 °C, using the IVIUM analyzer.

Monitoring Condition		L_1_ (PZT/BiT) 10 × 10 mm						L_3_ ((PZT/PZT) 10 × 10 mm					
Pristine ^AT*^	Frequency (kHz ±5%)	2.8	106.8	123.6	144.2	169.8	---	11.2	122.9	176.6	188.1	237.0	---
Pristine ^HT*^		2.8	105.5	---	140.7	---	---	10.2	120.2	---	184.3	---	---
Damaged ^AT^		2.6	102.1	112.5	132.3	155.8	171.7	6.0	116.7	167.3	179.5	225.8	537.5
Damaged ^HT^		2.6	99.7	---	127.3	---	---	4.8	109.5	137.5	141.8	173.3	---
Pristine ^AT^	EMI (Ω ±2%)	14800	168.5	145.1	131.3	125.5	---	3450	241	420.0	114.5	55.9	---
Pristine ^HT^		12880	134.4	---	106.9	---	---	3105	207	---	92.7	---	---
Damaged ^AT^		17400	184.8	173.9	148.4	138.2	124.3	4100	331	484.2	235.9	98.4	158.3
Damaged ^HT^		16320	161.4	---	122.1	---	---	4018	314	445.1	214.2	88.1	---

* AT: ambient temperature (20 °C), HT: High temperature (200 °C)

**Table 2:** Anti-resonance frequency and EMI of the blades L_2_ and L_4_ at 20 and 200 °C, using the transceiver circuit.

Monitoring Condition		L_4_ (PZT/BiT) 10 × 10 mm					L_2_ (PZT/PZT) 10 × 10 mm						
Pristine ^AT*^	Frequency (kHz ±5%)	3.0	98.5	116.4	158.4	191.8	2.9	4.0	13.9	---	---	---	---
Pristine ^HT*^		2.9	97.8	---	159.0	---	2.7	3.5	13.0	---	---	---	---
Damaged ^AT^		2.6	96.0	116.0	156.1	178.2	2.7	3.3	13.6	19.0	115.0	170.3	525.9
Damaged ^HT^		2.6	94.8	---	139.9	---	2.6	3.3	11.1	---	106.0	170.4	---
Pristine ^AT^	EMI (Ω ±2%)	12723	180.4	147.5	129.6	119.6	4864.4	2407.6	2399.0	---	---	---	---
Pristine ^HT^		11580	151.7	---	111.5	---	4163.1	2373.5	1144.8	---	---	---	---
Damaged ^AT^		13486	204.1	188.0	152.5	108.0	8402.3	2491.5	1158.1	731.2	427.4	516.4	173.7
Damaged ^HT^		13024	190.5	---	131.2	---	7406.8	2483.2	1118.0	---	288.9	287.0	---

* AT: ambient temperature (20 °C), HT: High temperature (200 °C)

**Table 3:** EMI frequency shift of the pristine and damaged blades at 200 °C compared to ambient temperature, using IVIUM and the transceiver circuit.

Impedance Analyzer	Blade L_1_					Blade L_3_			
	Pristine		Damaged			Pristine		Damaged	
	*ΔF*	*@ F*	*ΔF*		*@ F*	*ΔF*	*@ F*	*ΔF*	*@ F*
**IVIUM**	0.0	2.8	0.0		2.6	−1.0	11.2	−1.2	6.0
	−2.3	106.8	−3.4		102.1	−2.7	122.9	−7.2	116.7
	−3.5	144.2	−5.0		132.3	−3.8	188.1	−29.8	167.3
								−37.7	179.5
								–52.5	225.8
	**Blade L_4_**					**Blade L_2_**			
**Transceiver**	−0.1	3.0	0.0	2.6		−0.2	2.9	−0.1	2.7
	−0.7	98.5	−1.2	96.0		−0.5	4.0	0.0	3.3
	+0.6	158.4	−16.2	156.1		−0.9	13.9	−2.5	13.6
								−9.0	115.0
								+0.1	170.3
